# Evaluation of the BIOSECURE Questionnaire in Patients Followed for Inflammatory Rheumatological and Gastrointestinal Diseases Through the Analysis of This Questionnaire

**DOI:** 10.3390/jcm14030687

**Published:** 2025-01-22

**Authors:** Myriam Beissat, Marion Geoffroy, Loïs Bolko, Ambre Hittinger, Morgane Bonnet, Guillaume Cadiot, Jean Hugues Salmon

**Affiliations:** 1 Rheumatology Department, University Hospital Center of Reims, 45 Rue Cognacq-Jay, 51092 Reims, France; mgeoffroy@chu-reims.fr (M.G.); lbolko@chu-reims.fr (L.B.); ahittinger-roux@chu-reims.fr (A.H.); jhsalmon@chu-reims.fr (J.H.S.); 2 Faculty of Medicine, URCA—University of Reims Champagne Ardenne, 51100 Reims, France; gcadiot@chu-reims.fr; 3 Pharmacy and Pharmacovigilance Division, University Hospital Center of Reims, 45 Rue Cognacq-Jay, 51092 Reims, France; morganebonnet@chu-reims.fr; 4 Gastroenterology Department, University Hospital Center of Reims, 45 Rue Cognacq-Jay, 51092 Reims, France

**Keywords:** BIOSECURE, therapeutic education, targeted therapy

## Abstract

Therapeutic education (TE) plays a central role in the management of chronic inflammatory rheumatic diseases and inflammatory bowel disease. The BIOSECURE questionnaire was developed and validated in 2012 to assess self-management and patient safety, initially in rheumatology. **Objectives**: The aim of our study was to assess the knowledge of patients followed in both rheumatology and gastroenterology regarding their treatment through the BIOSECURE questionnaire. The secondary objective was to identify factors associated with a low level of knowledge according to the BIOSECURE questionnaire. **Methods**: This was a descriptive observational study, conducted in a single center at the Reims University Hospital between January 2023 and April 2024. The population was divided into quartiles. Participation in therapeutic education (TE) included receiving brochures about their disease or treatment and/or participation in group or individual TE sessions. We compared the patients with the lowest scores to those with the highest scores. **Results**: The study population consisted of 312 patients, including 32.05% with rheumatoid arthritis (RA) and 29.81% with Crohn’s disease. In our population, 82.03% had participated in therapeutic education, which included a TE session and/or the distribution of brochures about their disease and/or treatment. The median [IQR] BIOSECURE score was 71.04/100 [IQR 61.77–81.9]. When comparing patients with a low BIOSECURE score (<61.77) to those with a high score (>81.9), univariate factors associated with a low score were older age (*p* = 0.02), less participation in therapeutic education (*p* = 0.01), shorter duration of targeted therapy (*p* = 0.01), and lower level of education (*p* < 0.05). Conversely, patients who had received therapeutic education had a higher BIOSECURE score (*p* = 0.01). There was no difference in BIOSECURE scores based on place of residence, location of patient follow-up, route of administration, or type of inflammatory disease. In a multivariate analysis with a model including age, TE participation, and duration of targeted therapy, the results remained significant (*p* < 0.05). **Discussion:** We were able to compare the results of our study with two other French studies previously conducted on the same population of 677 patients undergoing biotherapy for chronic inflammatory rheumatism. The median BIOSECURE score in those studies was 73/100. In the study by Rat AC, published in 2017, the population was divided based on their BIOSECURE questionnaire results into three groups; they compared high and low response levels. Similarly to our study, a lower educational level and unemployment were associated with a lower rate of correct responses. The same was true for the absence of therapeutic education (TE) or distribution of brochures. **Conclusions:** The analysis of the BIOSECURE questionnaire in our population provides a practical message: factors associated with a low BIOSECURE score include older age, lower educational level, recent initiation of targeted therapy, and lack of participation in therapeutic education. This population could be a priority target for TE in order to ensure treatment safety for these patients.

## 1. Introduction

Patients followed for chronic inflammatory rheumatological (CIR) or intestinal diseases (IBD) require comprehensive management, integrating both pharmacological treatments and clear information, as well as therapeutic education from the diagnosis and throughout the course of the disease [1,2].

For the past 20 years, the advent of targeted therapies has revolutionized the prognosis for patients with inflammatory rheumatism or inflammatory bowel disease, significantly improving their condition [1,2,3,4]. However, these treatments carry specific risks, particularly infectious risks [5,6]. Therefore, to mitigate this risk, a pre-treatment evaluation is recommended [7], including the updating of vaccination records, as well as regular follow-ups with a dentist and dermatologist [5].

Patients with these conditions often have multiple comorbidities, which can have a potential impact on important clinical characteristics, such as treatment adherence, their engagement in care, and potential side effects.

The impact of therapeutic education for patients [8], to help them understand their pathology, treatment, and the potentially serious risks associated with it, is a central part of their management to promote self-management in terms of safety [9]. Patient education can be implemented through various methods: distribution of brochures about the disease and/or treatments and participation in individual or group therapeutic education sessions.

Given the serious potential side effects of targeted treatments, a questionnaire titled BIOSECURE was developed and validated in 2012 by the French Society of Rheumatology. The aim of the questionnaire is to assess self-management and patient safety, specifically in patients undergoing biotherapy, and thus provide an overview of our patients’ knowledge [10].

The objective of our study was to assess the knowledge of patients followed in both rheumatology and gastroenterology at our hospital regarding their treatment through the BIOSECURE questionnaire.

The secondary objective was to identify factors associated with a low level of knowledge as per the BIOSECURE questionnaire.

## 2. Materials and Methods

### 2.1. Study Design

This is a descriptive observational study, conducted in a single center at the Reims University Hospital between January 2023 and April 2024.

The data were obtained from the medical records of patients who filled out the BIOSECURE questionnaire as part of the follow-up of their targeted therapy for CIR or IBD.

### 2.2. Study Population

Eligible patients for our study were over 18 years old and required to be under targeted therapy for a chronic inflammatory disease such as IBD or CIR.

Patients were required to have completed the BIOSECURE questionnaire as part of their follow-up during consultations or hospitalizations.

### 2.3. Data Collection

#### 2.3.1. BIOSECURE Questionnaire 1

This questionnaire consists of 52 to 55 items depending on the route of administration of the patient’s targeted treatment, with a final score out of 100. The closer the score is to 100, the better the knowledge. It includes 29 knowledge-related questions, with 7 situational questions covering 7 domains: infection, vaccination, pregnancy planning, treatment storage, surgery planning, dental care, and treatment adherence (Table 1).

The questionnaire includes multiple-choice questions and 2 open-ended questions in the case of subcutaneous treatment. It was initially designed for the evaluation of patients with chronic inflammatory rheumatism, but we adapted it to fit the follow-up of patients with inflammatory bowel disease, particularly by changing the terms “rheumatism” to “inflammatory bowel disease” in certain situational questions.

This BIOSECURE questionnaire does not include oral treatments such as Janus kinase inhibitors, so we added this category in the first question regarding the patient’s targeted treatment and its oral administration route.

#### 2.3.2. Other Collected Data

Medical history and comorbidities (used to calculate the CHARLSON score [11]), demographic data (age, sex, education level, smoking status), clinical data (type of chronic inflammatory disease, duration of targeted therapy, follow-up location), treatment type, duration, and route of administration, vaccination status, recommended follow-up visits, and the presence of infectious events (requiring hospitalization or multiple antibiotic treatments) were recorded from the medical records.

The concept of therapeutic education for the patient, including either the distribution of brochures about the disease and/or the treatments or education by medical or paramedical staff in individual or group sessions, was also collected.

Additionally, we collected other self-reported questionnaires regarding Patient Reported Outcomes (PRO), such as the HAD score [12] (assessing anxiety and depression), and the EPICES score [13] (assessing vulnerability).

### 2.4. Statistical Analysis

We divided our population into quartiles. We compared patients with the lowest scores (Group 1) to those with the highest scores (Group 4) on the BIOSECURE questionnaire.

Regarding quantitative variables, we expressed them as medians with interquartile ranges. Qualitative variables were described with their frequencies and corresponding proportions.

Next, to examine the association between two qualitative variables, we performed a Chi-squared test. For comparing quantitative data, we used *t*-tests or Mann–Whitney tests depending on the normality of the data distribution. Finally, we performed a multivariate logistic regression analysis.

Values of *p* < 0.05 were considered statistically significant. Analyses were performed using R++ software, version 4.4.2.

### 2.5. Ethics

The data were collected from medical records, and they were protected and anonymized in accordance with French and European legislation, following the usual follow-up protocols.

Each patient was informed about the study, the type of data collected, and the study’s objectives.

This study was conducted according to the applicable French legislation and the methodology to which the Reims University Hospital adheres.

## 3. Results

### 3.1. Characteristics of the Population

The study population consisted of 312 patients with rheumatological or gastroenterological inflammatory diseases. Their characteristics are described according to the type of their inflammatory conditions (Table 2).

In our population, 82.03% of the patients had received therapeutic education, including either a therapeutic education session (51.39%) and/or distribution of brochures about their disease (65.37%) and/or treatment (52.44%).

### 3.2. Global BIOSECURE Analysis (Group 1 vs. Group 4)

**Figure 1:**
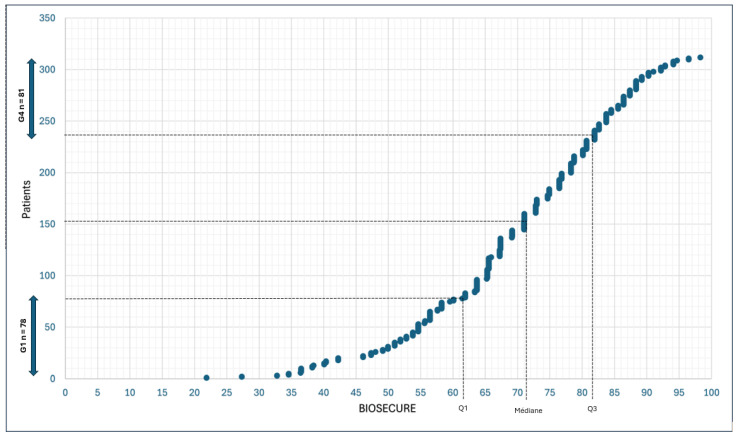
Scatter plot of the population based on the BIOSECURE score (0 to 100).

The distribution of the BIOSECURE questionnaire results in the 312 patients is represented in Figure 1.

In our study, encompassing both RA and IBD patients, the median [IQR] BIOSECURE score was 71.04/100 [IQR 61.77–81.9]. There was no significant difference in BIOSECURE scores across the different inflammatory diseases (*p* = 0.43).

We then compared the 25% of patients with the lowest scores (<61.77/100, *n* = 78) to the 25% of patients with the highest scores (>81.9/100, *n* = 81). Group 4 had more patients because several patients had scores close to 81.9/100 (Table 3).

Table 3 compared these two groups of patients to identify factors associated with a successful outcome on the BIOSECURE questionnaire, based on demographic and clinical criteria. Table 4 specifically addresses participation in therapeutic education in all its forms.

Significant findings include older patients (*p* = 0.02), less participation in therapeutic education (*p* = 0.01), a shorter duration of targeted therapy (*p* = 0.01), and lower education levels (*p* < 0.05) in Group 1. Only 24.66% of the patients in Group 1 had post-secondary education compared to 58.02% in Group 4.

There are significantly fewer retirees in the group with the higher BIOSECURE score (16.05% versus 41.03%, with *p* < 0.05).

If we look at the potential consequences for our patients of having a low score on this questionnaire, there is no significant difference between the two groups regarding the number of patients who were hospitalized for a severe infection or the number of antibiotic treatments per year. There is also no significant difference between the two groups regarding the updating of the vaccination schedule, across all vaccines.

No significant difference was found regarding the location of patient follow-up (*p* = 0.82) or the route of administration of the treatment (*p* = 0.55).

Additionally, no significant difference was found between the two groups regarding potential patient dissatisfaction with the control of their disease and their BIOSECURE score.

There is no statistical difference in the level of the BIOSECURE questionnaire between the different inflammatory pathologies.

Subsequently, we considered the impact of different types of therapeutic education on BIOSECURE scores (Table 4). It can be observed that all forms of therapeutic education are associated with better BIOSECURE scores. There is no significant difference based on the healthcare professional conducting the therapeutic education.

In multivariate logistic regression analysis, which included age, participation in therapeutic education, and the duration of biotherapy, the results remained significant (*p* < 0.05). Participation in therapeutic education was the factor most strongly associated with an improved BIOSECURE score, with the highest odds ratio at 3.39.

## 4. Discussion

As previously mentioned, the median [IQR] of the BIOSECURE score in our study is 71.04/100 [IQR 61.77–81.9].

By comparing patients with a low BIOSECURE score (Group 1) to those with a high score (Group 4), we were able to identify that patients with lower knowledge levels and therefore potentially greater safety risks are the older, retired patients who have been on targeted therapy for a shorter period and have not participated in any form of therapeutic education.

Patients with a higher education level had significantly better BIOSECURE scores.

In our study, among the 312 patients, there was no statistical difference in the number of hospitalizations between those with higher and lower BIOSECURE scores. Indeed, targeted treatments pose a significant infectious risk for our patients. Therefore, targeted therapeutic education on this point remains essential for all patients, regardless of whether they are in Group 1 or Group 4. A reminder about the infectious risk and the indication to suspend their treatment in the event of an infection should be emphasized after each hospitalization for a severe infection.

Therapeutic education (TE) is therefore essential to ensure that patients are well informed and understand their treatment, thereby improving their safety with treatments that are not without adverse effects, making them more comfortable with self-management.

Moreover, we demonstrated that the distribution of informative brochures to our patients was associated with the same BIOSECURE results as participation in in-person sessions. Thus, general practitioners who do not have easy access to TE sessions for their patients can provide brochures with similar outcomes.

A multicenter French study was conducted on 677 patients undergoing biotherapy, aiming to describe patient safety skills [14]. In comparison, the median score was 73/100 [60–82], which is similar to our population. This study, conducted in 2014, included only patients on IV or SC biotherapy, while our study also included 23 patients on oral treatments. The analysis of the questionnaire was based on different domains and expressed as percentages of correct answers. The domains requiring improvement were physician visits, vaccination, contraception, and the management of subcutaneous treatments.

A second study was conducted on the same population of 677 patients, with a goal similar to ours: to identify factors to target in order to improve patient care [15]. The questionnaire results were divided into three categories: high level (less than 9 incorrect answers), medium level (between 9 and 20 incorrect answers), and low level (more than 20 incorrect answers). They compared the high and low levels of responses.

As in our study, a lower educational level and being unemployed were associated with a lower percentage of correct responses. The same was true for the absence of TE or the distribution of brochures.

In their study, the authors also showed that living alone and residing in a large city were factors that favored a lower percentage of correct responses.

Unlike the previously mentioned studies [14,15], our study was conducted in a monocentric, tertiary care center where TE is offered to all patients; however, the results remain consistent with our study.

The main strength of our study is that the patient population corresponds to the typical group of patients followed in a rheumatology and gastroenterology service, with patients on intravenous, subcutaneous, or oral treatments. Indeed, this questionnaire has not been extended to other specialties with access to targeted therapies.

By using this questionnaire in routine practice, we were able to review with each patient their errors and gaps in knowledge regarding the safety of their treatment and provide relevant, targeted information for each patient.

The number of patients included in our study may account for the lack of statistical power observed for certain parameters. Another limitation was the evaluation of disease activity based solely on the patients’ own assessments, without any objective scoring or physician evaluation.

Another limitation of our study is that we used the first version of BIOSECURE (BIOSECURE 1) for its implementation, and since then, there has been an update that includes treatment with JAK inhibitors [16].

As shown in the results with the Charlson score, patients in the group with a lower BIOSECURE score have more comorbidities. Moreover, rheumatological diseases and IBD can be associated with immune and autoimmune disorders. However, we did not study the impact of associated diseases and potentially the treatments related to these conditions. The questionnaire does not assess the impact of associated diseases such as autoimmune hepatitis or celiac disease, for example [17,18].

All of these results highlight the need to offer therapeutic education to all our patients on targeted treatments, even if it is just the distribution of brochures. Through the BIOSECURE questionnaire analysis, we can identify patients who should be prioritized for TE: older patients, those with lower education levels, patients who have been on targeted therapy for a shorter time, and those who have never received therapeutic education. It will be important to enhance TE for these patients, as the implications of targeted treatments can affect their quality of life, infection risks, and the likelihood of hospitalization.

## 5. Conclusions

The analysis of the BIOSECURE questionnaire in our population provides a practical message:

- Factors associated with a low BIOSECURE score include older patients, those with a low level of education, patients on targeted therapy for a short time, and those who have not participated in any form of therapeutic education. This population can be a priority target for TE, ensuring their safety with treatment.

## Figures and Tables

**Table 1:** Description of the BIOSECURE 1 Questionnaire.

Categories	Domains/Competencies	Number of Items
Treatment Management		6
	Basic Knowledge	4
	Communication	2
Consultation with a Doctor		15
	Fever	11
	Infectious Symptoms	4
Specific Situations		19
	Vaccination	8
	Dental Care	2
	Surgery	7
	Contraception	2
Subcutaneous Treatment		3

**Table 2:** General characteristics of the study population and BIOSECURE results based on the type of their chronic inflammatory disease.

	RA 113 (36.22%)	Sp 86 (27.56%)	Crohn 93 (29.81%)	UC 20 (6.41%)	Total 312 (100%)
BIOSECURE/100					
Median [Q1, Q3]	72.8 [55.68, 82.56]	76.44 [64.08, 84.29]	67.34 [60.06, 76.44]	67.27 [61.43, 76.9]	71.04 [61.77, 81.9]
Age					
Median [Q1, Q3]	62 [52, 70]	51.5 [45, 59]	41 [31, 54]	30 [23.75, 36.75]	52 [40, 62]
Sex					
Female (%)	87 (76.99%)	42 (48.84%)	57 (61.29%)	13 (65%)	199 (63.78%)
Male (%)	26 (23.01%)	44 (51.16%)	36 (38.71%)	7 (35%)	113 (36.22%)
Marital Status #					
Single	16 (14.16%)	11 (12.94%)	18 (19.35%)	9 (45%)	54 (17.36%)
In a relationship	81 (71.68%)	70 (82.35%)	63 (67.74%)	10 (50%)	224 (72.03%)
Divorced	7 (6.19%)	3 (3.53%)	9 (9.68%)	1 (5%)	20 (6.43%)
Widower/widow	9 (7.96%)	1 (1.18%)	3 (3.23%)	0 (0%)	13 (4.18%)
Children ##	93 (83.04%)	64 (75.29%)	63 (67.74%)	6 (30%)	226 (72.90%)
Retired	59 (52.21%)	15 (17.44%)	11 (11.83%)	1 (5%)	86 (27.56%)
Methotrexate ##	52 (46.02%)	29 (34.52%)	7 (7.53%)	1 (5%)	89 (28.71%)
Corticosteroid ###	21 (19.09%)	7 (8.33%)	5 (5.38%)	5 (25%)	38 (12.38%)
Duration of targeted therapy (in years)					
Median [Q1, Q3]	9 [3, 15]	8 [5, 15]	7 [4, 10]	4 [2.75, 6.5]	8 [4, 13]
Administration					
IV	58 (51.33%)	45 (52.33%)	9 (9.68%)	4 (20%)	116 (37.18%)
Subcutaneous	37 (32.74%)	39 (45.35%)	83 (89.25%)	14 (70%)	173 (55.45%)
Oral	18 (15.93%)	2 (2.33%)	1 (1.08%)	2 (10%)	23 (7.37%)
Line of treatment ###					
First	33 (31.73%)	37 (49.33%)	42 (45.16%)	6 (30%)	118 (40.41%)
Second or +	71 (68.27%)	38 (50.67%)	51 (54.84%)	14 (70%)	174 (59.59%)
Disease control according to the patient ####					
Very well controlled	37 (33.33%)	30 (35.29%)	38 (40.86%)	3 (15%)	108 (34.95%)
Well controlled	59 (53.15%)	42 (49.41%)	37 (39.78%)	10 (50%)	148 (47.90%)
Moderately controlled	12 (10.81%)	10 (11.76%)	16 (17.20%)	2 (10%)	40 (12.94%)
Poorly controlled	1 (0.90%)	2 (2.36%)	1 (1.08%)	3 (15%)	7 (2.27%)
Not controlled at all	2 (1.80%)	1 (1.18%)	1 (1.08%)	2 (10%)	6 (1.94%)
CHARLSON score					
Median [Q1, Q3]	2 [1, 4]	1 [1, 2]	1 [0, 1]	0 [0, 1.25]	1 [0, 3]
Classes of medications #					
Anti TNF	34 (30.36%)	77 (89.53%)	54 (58.06%)	9 (45.00%)	174 (55.95%)
Anti CTLA4	21 (18.75%)	0 (0.00%)	0 (0.00%)	0 (0.00%)	21 (6.75%)
Anti CD20	5 (4.46%)	0 (0.00%)	0 (0.00%)	0 (0.00%)	5 (1.61%)
Anti IL12/23	0 (0.00%)	2 (2.33%)	30 (32.26%)	4 (20.00%)	36 (11.58%)
Anti IL17	0 (0.00%)	5 (5.81%)	0 (0.00%)	0 (0.00%)	5 (1.61%)
Anti IL6	34 (30.36%)	0 (0.00%)	0 (0.00%)	0 (0.00%)	34 (10.93%)
JAK inhibitors	18 (16.07%)	2 (2.33%)	1 (1.08%)	2 (10.00%)	23 (7.40%)
Anti IL 23	0 (0.00%)	0 (0.00%)	3 (3.23%)	0 (0.00%)	3 (0.96%)
Anti-integrins	0 (0.00%)	0 (0.00%)	5 (5.38%)	5 (25.00%)	10 (3.22%)

Note: Missing data: # out of 311; ## out of 310; ### out of 292; #### out of 309; + means more. Note 2: RA = rheumatoid arthritis; Sp = spondyloarthropathies (spondyloarthritis, SAPHO, psoriatic arthritis); UC = ulcerative colitis.

**Table 3:** Comparison of Group 1 and Group 4 Based on the BIOSECURE Score.

	Group 1 *n* = 78	Group 4 *n* = 81	*p*-Value (Univariate Analysis)	*p*-Value (Multivariate Analysis)
Age	54.13 (±19.46)	47.75 (±14.51)	0.02	<0.05
Therapeutic Education	56 (73.68%)	73 (90.12%)	0.01	<0.05
Duration of Biotherapy	7.22 (±5.32)	9.57 (±5.76)	0.01	<0.05
Sex			0.06	
Female (%)	47 (60.26%)	61 (75.31%)		
Family Medical Support	14 (18.42%)	12 (14.81%)	0.69	
Education Level			<0.05	
Primary/Secondary	3 (4.11%) #	2 (2.47%)		
High School	52 (71.23%) #	32 (39.51%)		
Post-secondary	18 (24.66%) #	47 (58.02%)		
Follow-up Location			0.82	
City (%)	3 (3.85%)	4 (4.94%)		
Hospital (%)	46 (58.97%)	44 (54.32%)		
Day Hospital (%)	29 (37.18%)	33 (40.74%)		
Route of Administration			0.55	
Intravenous (%)	31 (39.74%)	35 (43.21%)		
Subcutaneous (%)	43 (55.13%)	39 (48.15%)		
Oral (%)	4 (5.13%)	7 (8.64%)		
HAD			1.00	
Negative (%)	52 (68.42%) ##	55 (68.75%) ###		
Positive (%)	24 (31.58%) ##	25 (31.25%) ###		
CHARLSON Score			0.03	
Median [Q1, Q3]	2 [1, 3.25]	1 [0, 2.25]		
EPICES Score			0.11	
Vulnerable	29 (38.16%)	20 (25.00%)		

Note: data missing: # on 73; ## on 76; ### on 80.

**Table 4:** Comparison of Group 1 and Group 4 based on type of therapeutic education received.

	Group 1 *n* = 78	Group 4 *n* = 81	*p*
Brochures on Disease (%)	41 (53.95%) #	61 (76.25%) ##	<0.05
Brochures on Treatment (%)	28 (36.84%) #	52 (65.82%) ###	<0.05
Therapeutic Education Sessions (%)	30 (44.12%) ####	48 (61.54%) #####	0.05

Note: data missing: # on 76; ## on 80; ### on 79; #### on 41; ##### on 78.

## Data Availability

Data are available upon reasonable request.
